# Sustained Plus Lens Defocus Alters Retinal Responses and Ciliary Muscle Morphology In Myopes and Near-emmetropes

**DOI:** 10.1007/s44402-026-00169-2

**Published:** 2026-08-15

**Authors:** Sandra Wagner, Carina Sommer, Sophia Häupler, Kerstin Woll, Torsten Strasser

**Affiliations:** 1 Herbert Wertheim School of Optometry and Vision Science, University of California, Berkeley, California USA; 2 School of Engineering Sciences, Department of Applied Physics, AlbaNova, Royal Institute of Technology, Stockholm, Sweden; 3 Institute for Ophthalmic Research, University of Tuebingen, Tuebingen, Germany; 4 Eye Hospital Tuebingen, Tuebingen, Germany

**Keywords:** Ciliary muscle, Electroretinography, Myopia, ON/OFF pathway, Positive defocus

## Abstract

**Purpose:**

The origin of myopia is multifactorial, with nearwork as one possible risk factor. Spectacles with peripheral positive defocus aim to inhibit further ocular elongation. However, myopic eyes of young adults were found to become longer with positive defocus. Additionally, they showed reduced ability to use chromatic cues for detecting the retinal defocus sign. Possible underlying retinal differences to non-myopes are yet to be determined.

**Methods:**

The ciliary muscle (CM) was imaged in near-emmetropes and myopes (18–30 years of age) using optical coherence tomography after consecutively watching a movie for 30 min (i) in focus and (ii) with +3.00 D lenses, to assess possible interactions of defocus with accommodation. After a 2-h break, long flash electroretinograms (ERG) were measured after clear and defocused viewing. ERG b- and d-waves served as proxies to evaluate retinal ON- and OFF-responses. At a separate visit for a subgroup, the ERG experiment was exploratively repeated under red-light restriction.

**Results:**

B-wave amplitudes were reduced (−4.74 µV) and delayed (+0.30 ms, both *p* < 0.001) in both groups after experiencing defocus. The CM became significantly thinner in myopes only (−19.43 µm, *p* = 0.007). Exploratory outcomes with red filters revealed smaller b-wave amplitudes with defocus (Δ = −6.44 µV, *p* = 0.003), as well as delayed b-waves without (Δ = 1.03 ms) and with defocus (Δ = 0.67 ms, both *p* < 0.001) in both groups. OFF-response proxies of the myopic sub-group showed reduced i-waves after defocus, independent of light conditions.

**Conclusions:**

Sustained positive defocus reduced physiological proxies for retinal ON activity irrespective of the refractive error. After defocus exposure, the CM of myopes reacted similarly to after sustained nearwork, as found previously. Altered OFF-response proxies in myopes after blur could point to modified retinal processing. Paediatric trials are required to assess whether these acute physiological responses following fixed-sequence defocus exposure relate to ocular growth signals, myopia progression or optical treatment response.

Key Points

- This study found that prolonged plus lens wear induces acute changes in both the retina and ciliary muscle, having implications for future studies of myopia development and treatment in children.
- Only in myopes, the ciliary muscle, essential for accommodation, changes similarly after sustained blur and reading, supporting further investigation of the relationship between nearwork and myopia.
- The retina of young adults adapts to sustained plus lens wear, warranting investigation in children to determine potential interactions with optical myopia control treatments.

## Introduction

In the postnatal period, the vertebrate eye changes its length to match the optical power so that it maintains clear vision of distant objects. Animal models revealed that this process, termed emmetropisation, is visually controlled using the defocus signal on the retina [1]. However, in a myopic eye, the negative feedback loop of emmetropisation is disrupted, leading to excessive elongation of the eye [2]. Increased time spent indoors, as well as extensive close work, have been suggested as risk factors for myopia development [3]. Although the relationship between myopia and nearwork is controversial [4], it appears that the ciliary muscle (CM) is involved in several theories on myopia development [5–8].

To date, a plethora of interventions to inhibit myopia progression have been developed [9]. These span from pharmacological to light therapy [10] or optical treatments [11]. Regarding the latter, the mode of action is primarily based on positive defocus. By placing the image plane in front of the retina, stimulation for further eye growth was removed in animal models of myopia [12–15]. Also, reduced optical axial length with imposed positive defocus was found in humans [16]. Recently, a remarkable difference between short-term axial length changes with positive lens defocus was revealed in emmetropic vs. myopic young adults. Myopes, rather than responding with the expected shortening of axial length, developed temporarily longer eyes [17]. Several, partly yet unidentified visual cues might help in deciding whether the image lies in front of or behind the retina, e.g., monochromatic lower and higher order aberrations, such as spherical aberration or coma [1, 18]. It was suggested that longitudinal chromatic aberration under broadband light likewise adds important information since the eye focuses red (long wavelength light) behind shorter wavelengths, such as blue light [19]. However, conflicting results have been received in different animal models of myopia: Studies of rhesus monkeys [20] and tree shrews [21] revealed that red light reduced myopia development. Contrarily, chicks developed myopia when they were reared under red light, but hyperopia in blue light conditions [22]. In humans, 1 h exposure to either red or green light induced axial elongation, whereas exposure to blue light led to shorter eyes [23]. Interestingly, opposite axial length changes to plus lenses were found in myopes compared with emmetropes when the eye was deprived of chromatic information. In accordance with previous animal studies [20], Swiatczak and Schaeffel applied narrow-band interference filters of 620 nm to limit the spectral range. After having watched a movie with imposed plus lens defocus and red filters, emmetropic eyes became temporarily shorter, while myopic eyes became longer [24]. It was suggested that substantial functional changes in the retina of myopes might cause failure of emmetropisation [25]. Electrophysiological studies revealed that the human retina has a decoding system for optical defocus, with the paracentral retina being more reactive than the central area [26, 27]. The retinal area around 6–10° was found to detect positive defocus without any information from the fovea [28]. To help clarify whether visual processing depends on the eye’s refractive error, we suggest studying retinal activity changes after a period of adaptation to positive defocus in myopes and nonmyopes. A possible influence on the retinal ON and OFF system is of particular interest. The division into these two distinct pathways, which begin in the retina, helps the visual system process light increments and decrements in an energy- and space-saving manner [29]. The refractive development of animal models was found to be affected when ON/OFF channels were selectively blocked or activated [30, 31]. In human eyes, axial length changes can likewise arise after overstimulation of either pathway by changing the contrast polarity of reading text. Previous studies with young adults showed that white text on a black background predominantly stimulated the ON pathway and induced temporarily shorter eyes, while OFF activation (black text on white) resulted in longer eyes [32, 33].

It is important to understand the effects of defocus, as used in current myopia control contact and spectacle lenses, on retinal processing. Indeed, it is also not yet understood how defocus might affect the CM’s shape and behaviour during accommodation and whether this differs between myopic and nonmyopic eyes. Gaining insight into possible interactions between lens defocus and accommodation is vital, given that children and young adults spend extended time viewing digital devices or computers at close distances, either for school or during leisure time [34, 35].

The primary outcomes of this study are the changes in CM thickness (CMT) and changes in amplitude and latency of physiological proxies for retinal ON-/OFF-responses following prolonged positive lens defocus in near-emmetropic vs. myopic eyes. We hypothesise that both the CM morphology and retinal responses of myopes, compared with near-emmetropes, will exhibit reduced or opposite changes after 30 min of plus lens defocus. The secondary, exploratory outcome is changes in retinal ON-/OFF-response proxies without and with plus lens defocus during long wavelength light exposure in near-emmetropic vs. myopic eyes, i.e., to test whether the effects on the retina are influenced by the spectral light range [20, 24]. This study design allows the investigation of visual pathways and comparing retinal responses with ocular biometric data following a fixed-sequence focus-defocus exposure. The acute physiological changes identified in this study could initiate related clinical assessments in children, potentially leading to enhanced or novel methods of optical myopia control.

## Methods

### Subjects

Participants with either near-emmetropic or myopic refractive state (spherical equivalent refractive error (SER) measured by subjective refraction; near-emmetropic: SER ≥−0.50 and ≤0.50 D; myopic: SER <−0.50 and ≥−6.00 D) between the ages of 18–30 years were recruited among the students of the University of Tuebingen, Germany. The study adhered to the Declaration of Helsinki and was approved by the Ethics Committee of the Faculty of Medicine, University of Tuebingen (155/2022BO2). Volunteers were informed about the study’s procedures, data processing and their rights as participants. Informed consent was obtained from all subjects before starting the measurements. A priori power analysis (power 0.8; alpha 0.05; effect size 0.5) indicated a required sample size of 34 individuals.

### Experimental Procedure

**Fig. 1:**
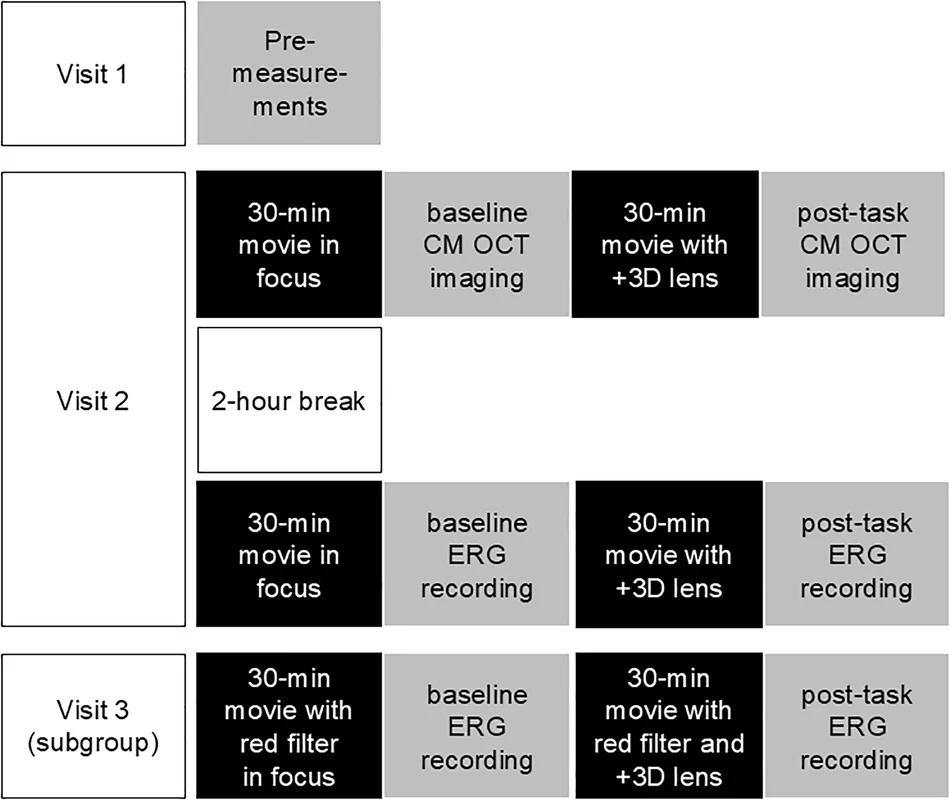
Experimental protocol. Description of the measurements and procedures during the study visits. The order of in-focus and defocus sessions during visits 2 and 3 was fixed and not counterbalanced. CM ciliary muscle, ERG electroretinograms, OCT optical coherence tomography.

Pre-measurements were performed in interested volunteers to test eligibility. Inclusion criteria were corrected monocular visual acuity of Snellen 6/6; SER between −6.00 and +0.50 D; astigmatism ≤2.00 D; age 18–30 years and no eye disease. Measurements included an ophthalmological screening for eye health (anterior segment and fundus biomicroscopy), medical history taking, objective refraction (Nidek AR-360A, NIDEK Co., Ltd, nidek-intl.com) and subjective refraction with monocular and binocular best-corrected distance visual acuity testing. Measurements of intraocular pressure (IOP, iCare IC-100, Icare Finland Oy, icare-world.com) and accommodative amplitude (Δ*A*_max_; push-up method) were also completed. Suitable candidates were invited to return for the main measurements. The experiment was undertaken within 1 day, with CM assessments taking place in the morning and electroretinography (ERG) after a break of 2 h to ensure sufficient time for relaxation and prevent fatigue. A subgroup of the participants agreed to return for a third visit, during which ERG assessments only were performed with additional red filter usage (Fig. 1). Myopes were corrected with soft contact lenses for clear distance vision during all measurements. Room illuminance during the entire experiment was maintained at about 40 lx.

### CM Imaging

First, the participants binocularly watched a coloured movie for a continuous period of 30 min on a 65” organic light-emitting diode (OLED) screen (LG OLED65B29LA OLED TV, LG OLED, 4K, 3840 × 2160 pixel; LG Electronics Deutschland GmbH, lg.com) at a distance of 2.00 m (without any monitoring of accommodation during the exposure). After this 30-min period, the right eye’s CM was imaged using an anterior segment optical coherence tomography (AS-OCT) instrument (Visante, Carl Zeiss Meditec AG, zeiss.com/meditec-ag/de/home.html). CM imaging was performed using the setup and protocol described previously [36]. In brief, subjects were seated in front of the OCT and their heads were centrally aligned to the device. Subjects were asked to shift their gaze to a high-resolution display (Adafruit Qualia 9.7” DisplayPort Monitor, Adafruit.com) at an eccentricity of approximately 40°. This display, positioned at a distance of 25 cm, was equipped with a semi-transparent mirror so that subjects could align the near screen with a second display, presented at a distance of 4.00 m. This setup was chosen to reduce eye movements during the change from one display to the other. After individual alignment, which took about 2 min, the right eye’s CM was imaged, while subjects fixated the target (5-letter words) with their left eye. Imaging started with the presentation of words on the far display (relaxed accommodation). After having obtained a minimum of three OCT images, the target presentation switched to the near display. Subjects were asked to accommodate to keep the presented word clear. Additional eccentric infrared photorefraction recordings were obtained to confirm that subjects were changing their refractive power appropriately for both viewing distances. After OCT imaging, +3.00 D trial lenses (in a trial frame) were placed before both eyes, while participants were seated in front of the OLED screen at a 2.00 m distance to watch the same movie for 30 min. The lenses produced around +2.50 D of relative defocus on the retina [17]; however, absolute values for each individual may vary due to any residual refractive error or changes in contact lens centration. The movie-watching with plus lenses was followed by further CM imaging in both relaxed (0.00 D) and accommodated (4.00 D) states, following the same protocol as before. Subsequently, participants were given a 2-h break.

### ERG Measurements

ERG was performed under light-adapted conditions using a visual electrophysiology recording system (Espion e², Diagnosys LLC, diagnosysllc.com) with a full field stimulator (ColorDome, Diagnosys LLC, diagnosysllc.com). After cleaning the skin with a peeling paste (Everi, Spes Medica S.r.l., spesmedica.com) to reduce impedance, a ground electrode was placed on the forehead and a counter electrode on the right temple. A Dawson, Trick, Litzkow (DTL) electrode [37] was inserted along the right eye’s lower eyelid below the corneal limbus. Subjects were asked to sit in front of the OLED screen to watch the same movie as earlier that day, again for a period of 30 min, first without plus lenses. While participants were watching the movie, the DTL electrode was temporarily removed and attached to the subject’s cheek. This in-focus episode was followed by the baseline ERG measurements, for which the DTL electrode was reinserted, which, together with another impedance check, took about 2 min. Using the stimulator’s ganzfeld field, an On–Off ERG [38] was recorded from the subjects’ right eyes without pupil dilation (stimulus length 500 ms; inter sweep delay 100 ms; sampling frequency 1000 Hz; luminance 30 vs. 330 cd/m²; two repeats of 20 sweeps each). Subsequently, subjects watched the same movie with additional +3.00 D lenses in front of both eyes for a further 30 min. Again, the DTL electrode was removed and attached to their cheek to increase subject comfort. After the 30 min viewing, the electrode was placed back into the lower eyelid, and another On–Off ERG was recorded using the same protocol as before. For both sessions, the order of the conditions was fixed and not counterbalanced, always starting with watching the movie in-focus (Fig. 1).

While the original aim was to recruit all participants for a second, exploratory part of the study, unfortunately, only some of them were willing to return due to time constraints. During this third visit, only the ERG was repeated in the same manner as before. However, during both watching periods, i.e., without and with +3.00 D lenses, narrow-band red filters (620 ± 10 nm, Edmund Optics GmbH, edmundoptics.com) were placed in the trial frames in front of both eyes. To prevent light entering the eye from above or the sides, subjects wore a cap with blinders during these trials.

### Data Processing

Images of the CM were processed using our previously developed semi-automatic software for a detailed analysis of the muscle’s morphological structure [39]. CMT was analysed at the position of the CM apex (CA). Additionally, the software allowed the evaluation of continuous CMT profiles along the muscle’s border plotted against the distance from the scleral spur (SP), a strong ring of elastic and collagen fibres [40]. As previously explained in detail [39], the SP serves as the origin of this coordinate system. For all measures, three OCT images were taken per subject and accommodative state (0.00, 4.00 D), and averaged for the without and with defocus conditions.

ERG recordings were pre-processed (Bandpass filter 0.312 to 300 Hz) and manually cleaned from artefacts arising from blinks (5.9% in the myopic group; 8.0% in the near-emmetropic group) following the International Society for Clinical Electrophysiology of Vision (ISCEV) standards. Such artefacts were easily detectable as non-physiological rapid amplitude changes. Artefacts that corresponded in shape to the responses created by the photomyoclonic reflex [41] were also excluded. Pre-processing of individual sweeps was performed manually due to the use of custom ERG protocols programmed in Python (python.org), which did not allow for automatic sweep rejection and repetition. Subsequently, using the electrophysiology system’s software (Espion Multifocal V6.0.54, Diagnosys LLC, diagnosysllc.com), markers for ERG a-, b-, c- and d-wave amplitudes were placed manually [38]. Rejection of individual sweeps as well as marker placement was performed by one of two experienced researchers in an unmasked manner for several consecutive batches following the sequence of measurements, and was double checked by a second experimenter. The b-wave is followed by a negative potential, the photopic negative response (PhNR), which derives from retinal ganglion cell (RGC) activity [42]. A positive potential, termed the i-wave, in the descending branch of the b-wave might reflect the activity of cells distal to the RGCs, but its exact origin remains to be determined [43]. With longer flashes as used in this protocol, the PhNR and i-wave also appear after the d-wave (i_off_-wave) [44]. The onset- and offset-driven ERG components b- and d-wave are used as surrogates for ON and OFF pathway-weighted retinal activity, respectively. Their amplitudes can only serve as pathway-weighted proxies since isolation of retinal ON- and OFF-responses is not possible. It should also be noted that inner-retinal components like the PhNR and i-wave overlap with long flashes and are difficult to be separated clearly.

Data were exported for further processing, which was necessary to allow statistical analysis. The ERG b- and d-waves were assessed using their amplitudes and latencies according to ISCEV standards. In contrast, the i-wave and PhNR were evaluated as part of the pointwise *t*-testing over the entire recording period [45]. To assess ERG response curves over time, On–Off ERGs were divided into ON- and OFF-responses, followed by additional baseline corrections of the OFF-responses: Using the raw data for each subject and condition, the average voltage of the first 10 ms of the ERG recording was calculated and subtracted from each sweep. Then, the average of the two repeats of On–Off ERGs for each condition and subject was calculated.

### Statistical Analysis

Statistical analysis of CMT and ERG data was undertaken using JMP software (JMP Statistical Discovery LLC, version 16, jmp.com). A linear mixed effects model (LMM) was applied to assess the dependent variable CMT, with the factors condition (focus/defocus), refraction (near-emmetropic/myopic) and distance (0.00/4.00 D), as well as their interactions. Subject was added as a random variable. ERG b-wave (proxy for ON-response) and d-wave amplitudes (proxy for OFF-response), as well as latencies, were analysed, applying LMMs with the factors condition (focus/defocus), refraction and their interactions, again with subject as a random variable. Having performed two repeats of the On–Off ERG, the factor result number was added to assess the repeatability.

ERG data from the exploratory experiment (narrow-band light conditions) were compared with the results obtained from the subgroup tested at the second visit (broadband light conditions). Amplitude and implicit times of ERG b- (proxy for ON-response) and d-waves (proxy for OFF-response) were analysed in LMMs with the factors: condition (focus (f)/defocus (d)/focus with red filter (rf)/defocus with red filter (rd)), refractive group and their interactions. Again, the result number (two repeats of 20 sweeps) was added as a factor and subject as a random variable. Two-tailed post-hoc tests (Student’s *t*-test; Tukey’s HSD) at a 0.05 significance level were applied.

In addition to the LMM analysis, the retinal response changes over the entire recording time were assessed by applying pointwise *t*-tests (alpha = 0.05). Further, 95% confidence intervals (CI) were used to compare ERG results of near-emmetropes vs. myopes (difference of means) and the focus vs. defocus conditions (visit 2 + 3; mean difference) and broadband vs. narrow-band light (visit 3; mean difference).

To identify statistically significant temporal clusters in the pointwise paired *t*-statistics, a Threshold-Free Cluster Enhancement (TFCE) analysis [46] was performed using a sign-flipping permutation test (5000 permutations, default parameters *E* = 0.5, *H* = 2.0), in which the null distribution was generated by randomly flipping the signs of the observed *t*-values; corrected *p*-values were assessed two-tailed at alpha = 0.05 [46].

## Results

### Subject Characteristics

A total of 20 near-emmetropic (SER right eye −0.11 ± 0.24 D; Δ*A*_max_ = 8.94 ± 1.99 D; 24.15 ± 2.96 years; 10 males) and 20 myopic (SER right eye −2.63 ± 1.22 D; Δ*A*_max_ = 9.58 ± 1.83 D; 23.75 ± 3.01 years; 6 males) young adults participated.

### Effects of 30 min of Plus Lens Defocus on CM Morphology

CMT analysis revealed a statistically significant effect of viewing distance. The CM was significantly thicker with 4.00 D accommodation than when accommodation was relaxed (*F*_1,114_ = 198.59; *p* < 0.001; Student’s *t*-test Δ_4 D-0 D_ = +58.71 µm, [50.45; 66.96]). Moreover, there was a significant interaction (*F*_1,114_ = 12.60; *p* < 0.001) between refractive error and condition focus/defocus. Post-hoc analysis revealed that the CM of myopes was significantly thinner after the defocus exposure (Δ_defocus-focus_ = −19.43 µm, [4.07; 34.79]; *p* = 0.007; Tukey’s HSD; 0.00 D: −20.83 µm; 4.00 D: −18.02 µm, SEM 8.33 µm). This thinning was not observed for near-emmetropes (Δ_defocus–focus_ = +10.15 µm, [−5.21; 25.51]; *p* = 0.32; Tukey’s HSD; 0.00 D: +10.55 µm; 4.00 D: +9.75 µm, SEM 8.33 µm).

**Fig. 2:**
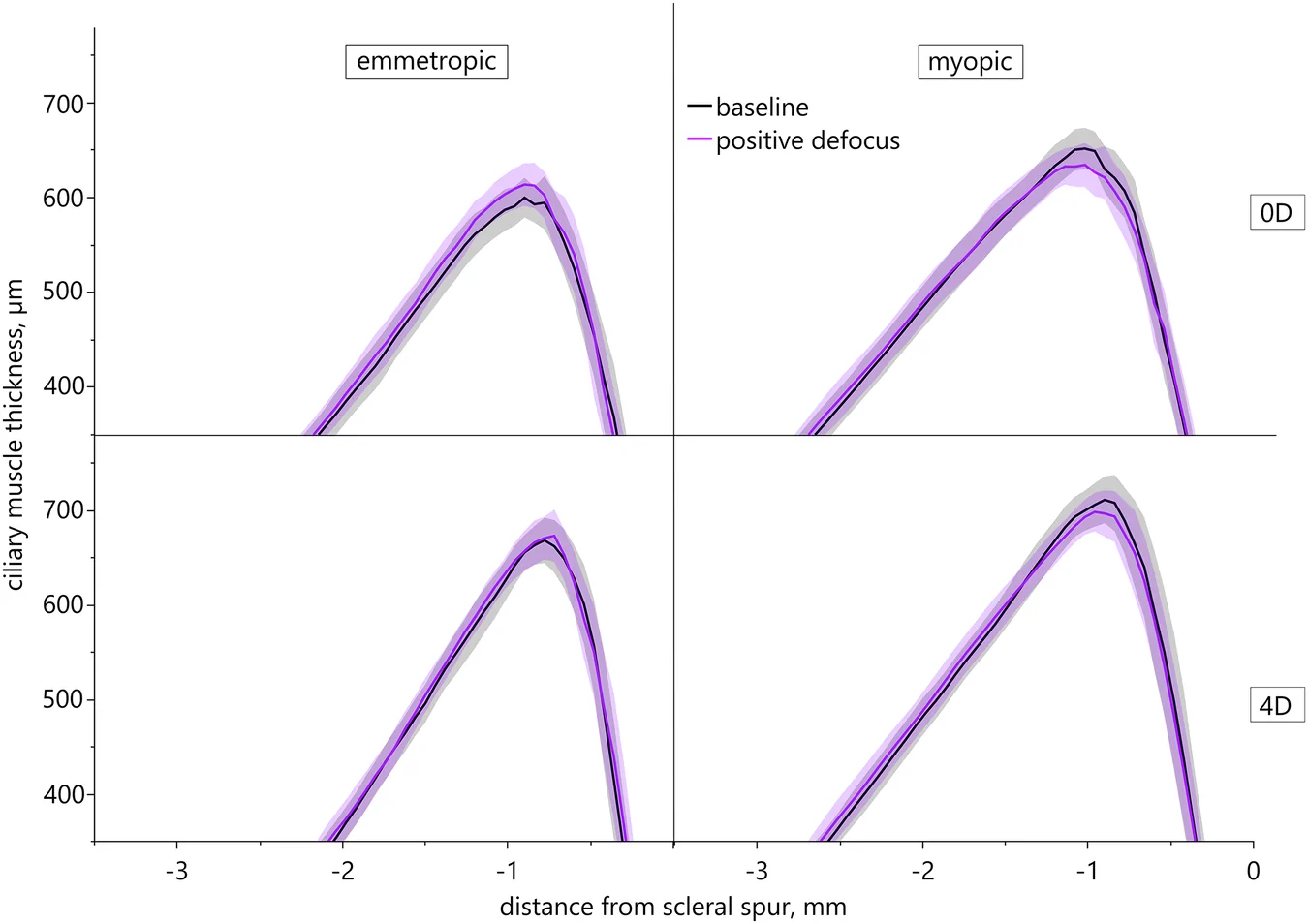
Ciliary muscle thickness profiles before and after exposure to plus lens defocus. Ciliary muscle thickness (CMT) profiles of near-emmetropic (left) and myopic subjects (right) at baseline (black line) and after 30 min of plus lens defocus (purple line). Profiles focus on the area of the largest change, 1 mm from the scleral spur. The upper graphs depict CMT during relaxed accommodation (0.00 D) and the lower graphs depict CMT when viewing a 4.00 D accommodation demand. Shaded areas denote ±1 SD.

Analysis of CMT measured along the CM boundary and plotted as a function of the distance from the SP showed CM thickening after exposure to defocus in near-emmetropic eyes, especially when accommodation was relaxed (Fig. 2, top left). To the contrary, the CM of myopes was thinner after the period of defocus for both viewing conditions, i.e., 0.00 and 4.00 D (Fig. 2, right column). However, these differences were not significant.

### Effect of Plus Lens Defocus on ERG Responses

LMM analysis (Table 1) revealed significantly reduced b-wave amplitudes after exposure to defocus, irrespective of the refractive error (*F*_1,117_ = 25.59; *p* < 0.001; −4.74 µV, [2.88; 6.60], SEM 0.94 µV; Student’s *t*-test). The reductions in amplitude were −6.19 µV in near-emmetropic and −3.29 µV in myopic eyes (both SEM 1.33 µV).

**Table 1:** Results of the linear mixed effects model of b-wave amplitude and implicit time.

	Df	*F*	*p*-value	Student’s *t*-test			
				Δ(defocus-focus)	SEM	CI	*p*-value
*b-wave amplitude*							
Refractive group	1,38	0.13	0.72				
Condition	1,117	25.59	<0.001	−4.74 µV	0.94 µV	2.88; 6.60	<0.001
Refractive group x Condition	1,117	2.39	0.13				
Result number	1,117	0.15	0.70				
*b-wave implicit time*							
Refractive group	1,38	1.21	0.28				
Condition	1,117	15.23	<0.001	0.30 ms	0.08 ms	0.15; 0.45	<0.001
Refractive group × Condition	1,117	0.11	0.75				
Result number	1,117	0.95	0.33				

*Df* degrees of freedom, *CI* confidence interval, *SEM* standard error of the mean.

The b-wave implicit times were increased significantly after the defocus period in both groups (*F*_1,117_ = 15.23; *p* < 0.001; +0.30 ms, [0.15; 0.45], SEM 0.08 ms; Student’s *t*-test). The respective mean increase in latency was +0.28 ms [−0.01; 0.56] in near-emmetropic and +0.33 ms [0.04; 0.61] in myopic retinae (both SEM 0.11 ms). B-wave amplitudes (*F*_1,38_ = 0.13; *p* = 0.72) and implicit times (*F*_1,38_ = 1.21; *p* = 0.28) did not differ significantly between near-emmetropes and myopes. Moreover, there was no significant interaction between refractive group and condition for amplitude (*F*_1,117_ = 2.39; *p* = 0.13) or implicit time (*F*_1,117_ = 0.11; *p* = 0.75) of this ON-response proxy.

The result for the OFF-response proxy (d-wave amplitude: *F*_1,117_ = 3.80; *p* = 0.05; implicit time: *F*_1,117_ = 0.40; *p* = 0.53) indicates weak/inconclusive evidence and should be interpreted with caution. Both did not differ significantly between the refractive groups (amplitude: *F*_1,38_ = 0.14; *p* = 0.71; implicit time: *F*_1,38_ = 0.005; *p* = 0.94). Also, the interactions between refraction and defocus did not show significant effects on the d-wave time (*F*_1,117_ = 0.18; *p* = 0.67) nor the amplitude (*F*_1,117_ = 1.92; *p* = 0.17).

**Fig. 3:**
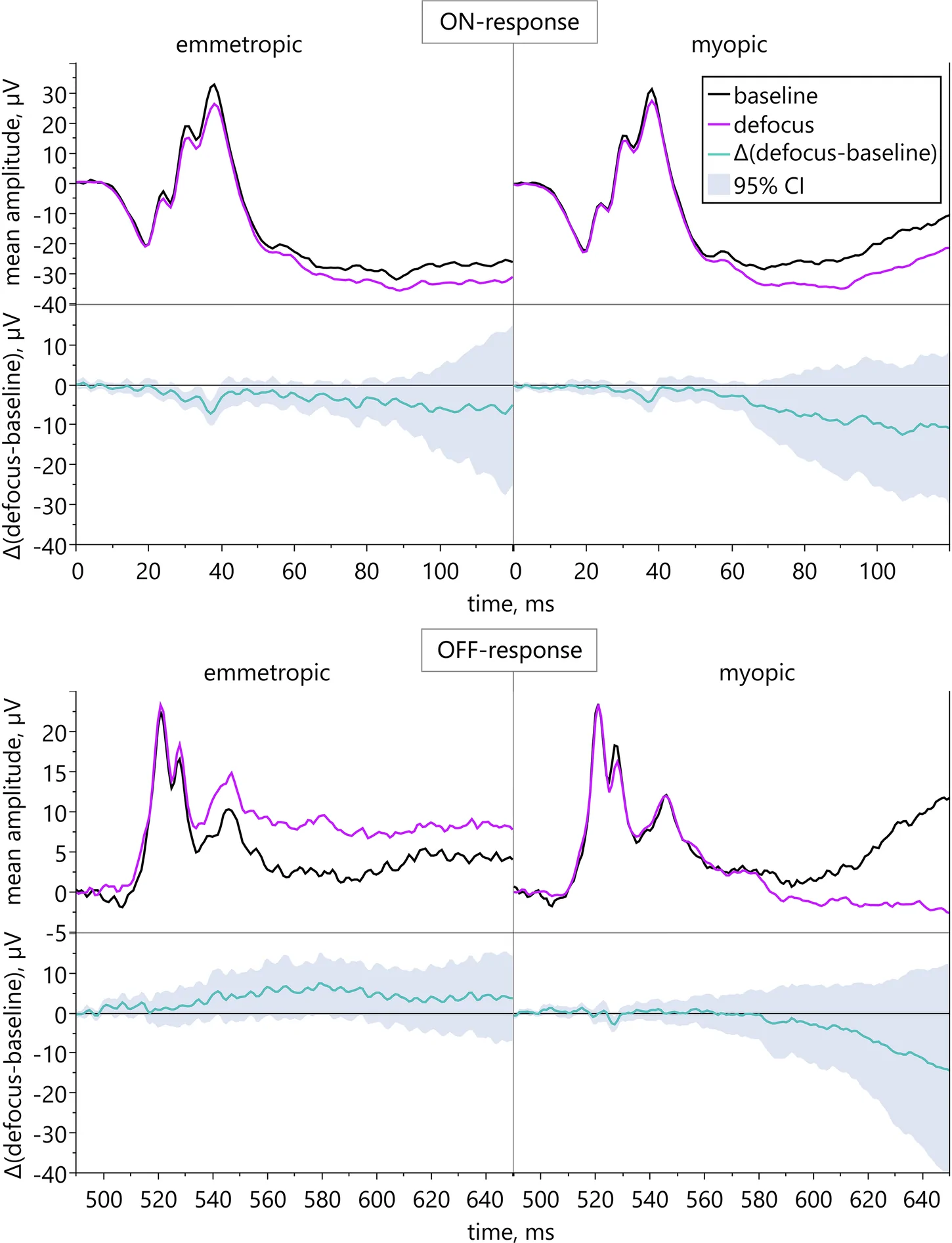
Retinal response changes of physiological proxies for the ON and OFF pathways after 30 min of plus lens wear. Physiological proxies for retinal ON- (top) and OFF-responses (bottom) of near-emmetropic and myopic eyes before (black line) and after (purple line) 30 min of exposure to defocus. The blue line in the lower graphs shows the mean difference Δ (defocus-baseline) with the shaded area depicting the 95% confidence intervals (CI).

**Fig. 4:**
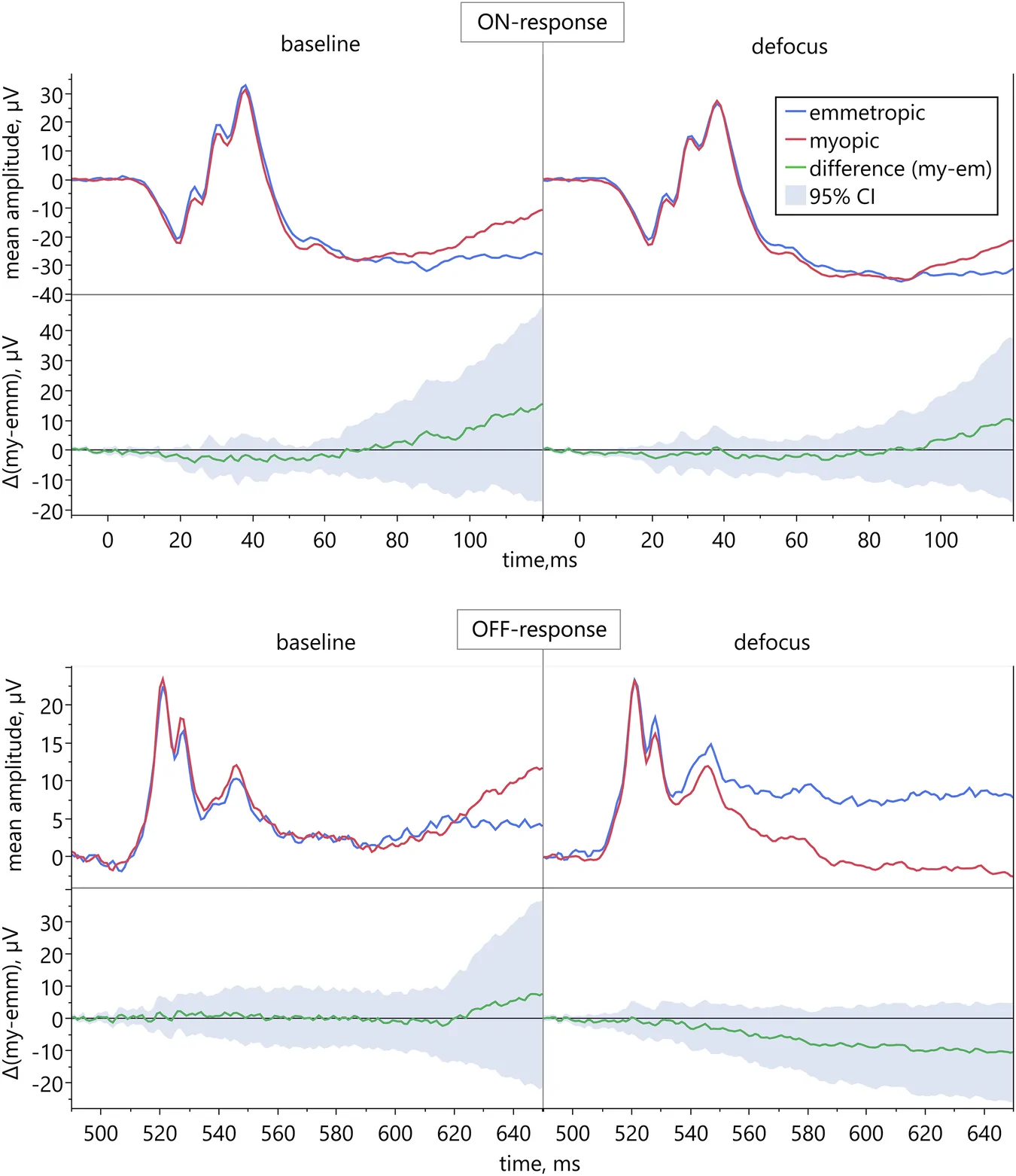
Electroretinogram (ERG) results of near-emmetropic (em) vs. myopic (my) subjects after 30 min of defocus. Physiological proxies for retinal ON- (top) and OFF-responses (bottom) of near-emmetropic (blue line) and myopic subjects (red line) before (left column) and after 30 min of exposure to defocus (right column). The green line shows the mean difference between groups (myopes–near-emmetropes) with the shaded area denoting the 95% confidence intervals (CI).

Assessment of ERG response curves using TFCE for both near-emmetropic and myopic subjects revealed a single significant timepoint in the physiological proxy for the ON-response at 37 ms post-stimulus onset (near-emmetropic: *t* = −4.93; myopic: *t* = −3.35; both *p* < 0.05, TFCE-corrected). The CI plots of the pointwise *t*-tests illustrate a significant reduction in the retinal ON-response proxies after 30 min of plus lens wear compared with baseline measurements in both refractive groups (Fig. 3, top). No significant changes in the retinal OFF-response proxy were revealed in either refractive group (Fig. 3, bottom). Nor were there any significant differences in ERG responses between near-emmetropic and myopic eyes (Fig. 4).

### Exploratory Outcome of Defocus Exposure Under Narrow-band Red Light Conditions

A sub-group of eight near-emmetropic (SER right eye −0.06 ± 0.21 D, Δ*A*_max_ 9.10 ± 1.87 D, 24.25 ± 2.60 years, three males) and 11 myopic (SER right eye -2.92 ± 1.28 D, Δ*A*_max_ 10.00 ± 2.18 D, 24.00 ± 3.61 years, four males) volunteers from the entire cohort participated in the third visit. It should be noted that the following results are exploratory and, unless confirmed by neutral-density controls, they may be confounded by spectral/illuminance differences. The periods of focus and defocus, experienced under red or broadband light (conditions: focus (f)/defocus (d)/focus with red filter (rf)/defocus with red filter (rd)), significantly affected the b-wave amplitudes (*F*_3,126_ = 7.41; *p* < 0.001). The amplitudes were largest for focus in red light and differed significantly between focus vs. defocus under both light conditions, broadband (Δ_f–d_ = 4.88 µV, [0.23; 9.52]; *p* = 0.04; Tukey’s HSD) and red light (Δ_rf–rd_ = 6.44 µV, [1.79; 11.08]; *p* = 0.003; Tukey’s HSD), as well as between red light focus vs. broadband light defocus (Δ_rf–d_ = 7.31 µV, [2.67; 11.96]; *p* < 0.001; Tukey’s HSD). The condition, i.e., focus (f)/defocus (d)/focus with red filter (rf)/defocus with red filter (rd), also had a significant influence on b-wave latencies (*F*_3,126_ = 18.163; *p* < 0.001). These differed significantly between broadband vs. red light for the focus and defocus conditions (Δ_rf–f_ = 1.03 ms, [0.58; 1.48]; Δ_rd–d_ = 0.67 ms, [0.22; 1.12]; both *p* < 0.001; Tukey’s HSD). Refractive error did not have a significant effect on b-wave amplitudes (*F*_1,17_ = 0.203; *p* = 0.66) nor latencies (*F*_1,17_ = 0.097; *p* = 0.76).

Amplitudes and implicit times of the physiological proxy for retinal OFF-responses, i.e., the d-wave, were not significantly changed with the various conditions (amplitudes: *F*_3,126_ = 2.281; *p* = 0.08; implicit times: *F*_3,126_ = 1.723; *p* = 0.17) nor dependent on the refractive error (amplitudes: *F*_1,17_ = 0.01; *p* = 0.92; implicit times: *F*_1,17_ = 0.07; *p* = 0.80).

**Fig. 5:**
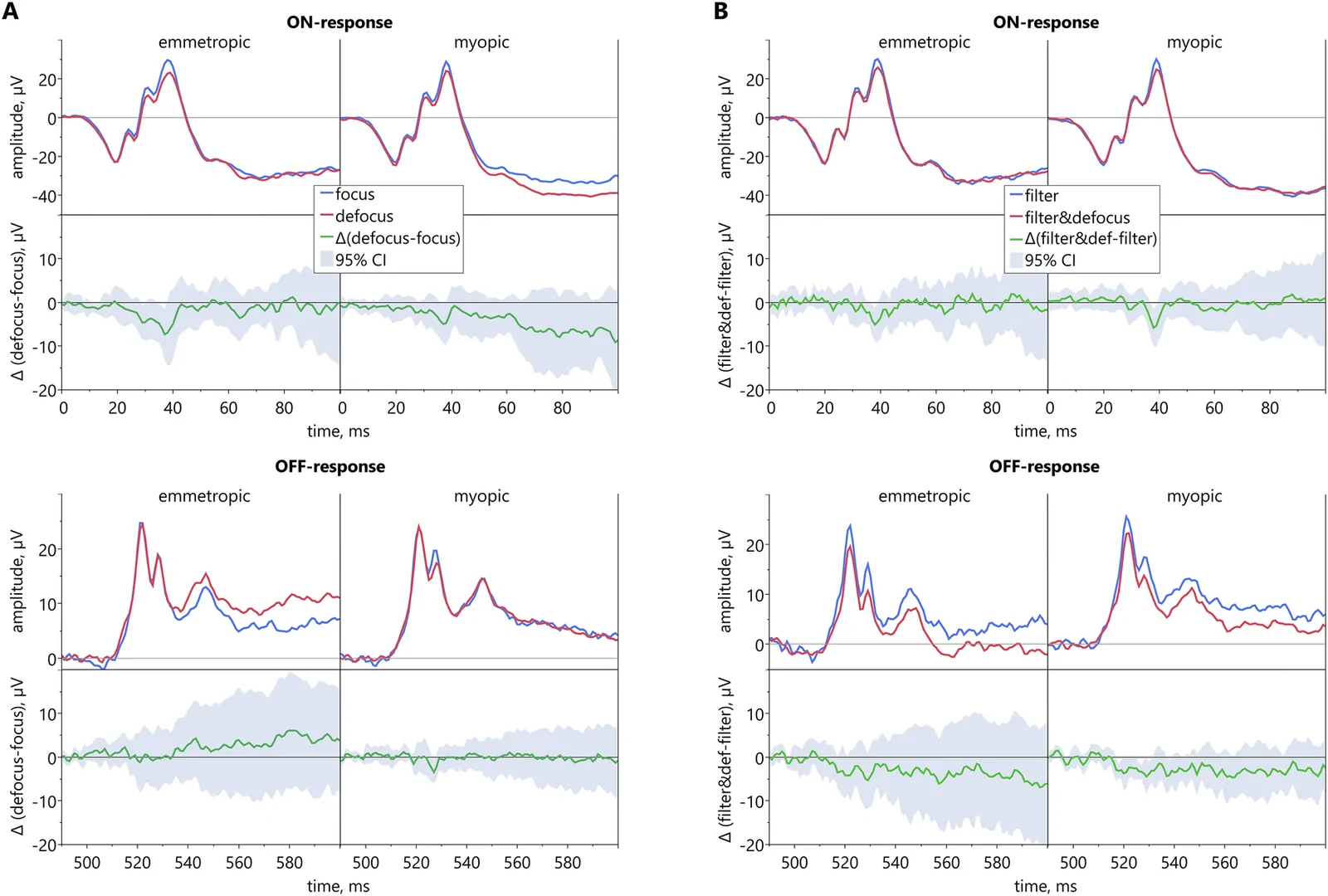
Pairwise comparison of exploratory studies of retinal responses under broadband and narrow-band light conditions. Physiological proxies for ON- and OFF-responses without (blue line) and with +3.00 D defocus (red line) under white (**A**) and red light conditions (**B**) in a subgroup of participants (*n* = 19). Defocus reduced ON-response proxies in near-emmetropic and myopic eyes without and with the red filter. Following defocus exposure, i-waves after stimulus offset (peak at ~530 ms) were reduced in myopic eyes. Lower graphs show the mean difference (green line) between defocus and clear conditions. Shaded areas: 95% confidence intervals (CI).

**Fig. 6:**
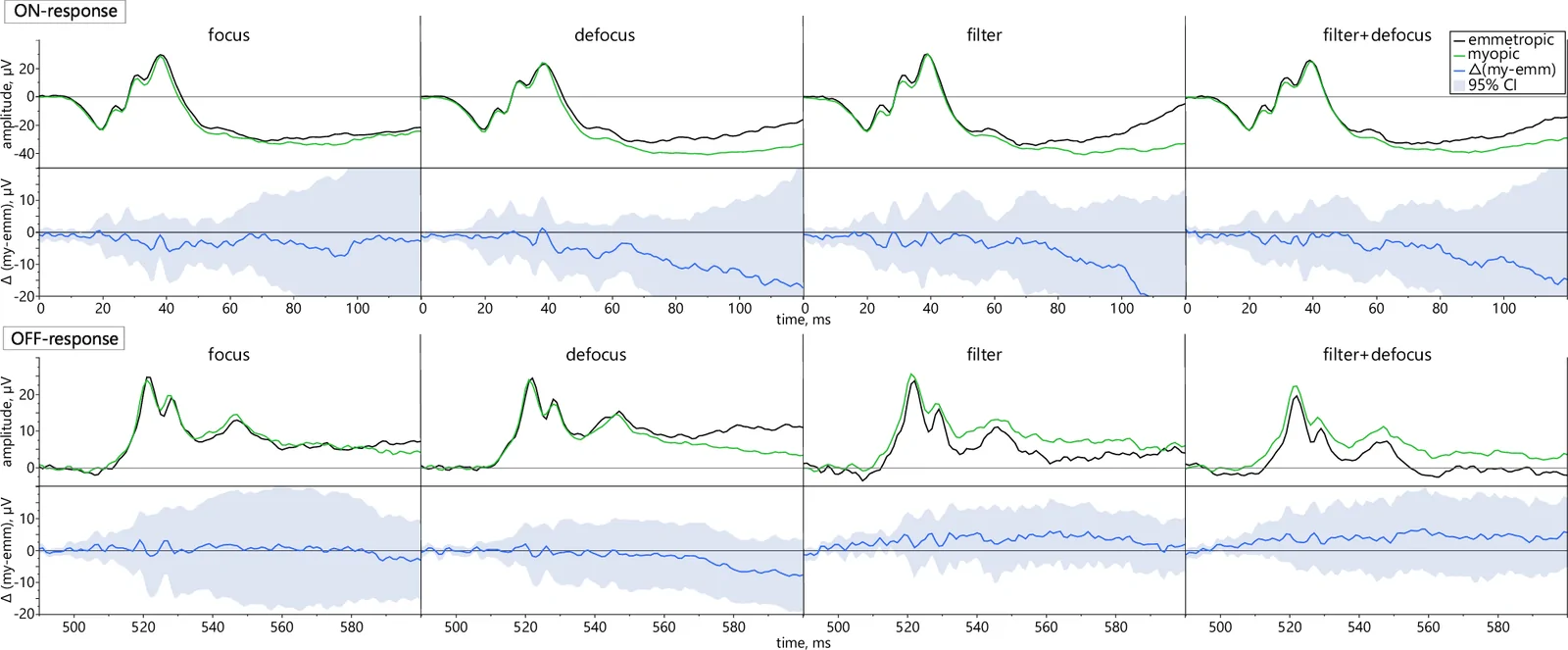
Pairwise comparison of exploratory studies of near-emmetropic vs. myopic retinal responses. Pointwise *t*-test of physiological proxies for retinal ON- and OFF-responses of myopic (green line) and near-emmetropic subjects (black line) for the four conditions: focus, defocus (both in broadband light), red filter and red filter with defocus. The lower graphs in the subplots show the mean difference (blue line) between the two refractive groups (myopic–near-emmetropic) with 95% confidence intervals (CI) (shaded areas).

Regarding the conditions in broadband light (Fig. 5A), TFCE analysis of the ERG recordings baseline vs. defocus identified two coherent timepoints at 36–37 ms (*t* = −2.93, *p* < 0.001) in near-emmetropes, as well as significant timepoints at 36–37 ms after stimulus onset (*t* = −2.41, *p* < 0.001) in myopes. For comparison of in-focus vs. defocused conditions under red light (Fig. 5B), for both refractive groups, TFCE revealed a single significant timepoint at 38 ms post-stimulus onset (near-emmetropic: *t* = −3.02, *p* < 0.001; myopic: *t* = −3.01, *p* = 0.005, TFCE-corrected). Interestingly, in myopes, TFCE analysis of ERGs after defocus exposure revealed a significant timepoint at 527 ms for broadband (*t* = −2.97, *p* = 0.01) and red light conditions (*t* = −4.88, *p* < 0.001), which refers to the area of the i-wave after stimulus offset (Fig. 5A and B). While the 95% CI plots of the pointwise *t*-test (alpha = 0.05) show that defocus reduced ON-response proxies significantly in near-emmetropic and myopic eyes, both without (Fig. 5A) and with the narrow-band red filter (Fig. 5B), no significant differences were observed between the two groups (Fig. 6).

## Discussion

This study investigated the effects of 30 min of fixed-sequence plus lens defocus on CM morphology and retinal activity in young adults with near-emmetropia or myopia. The results showed that sustained defocus affected retinal activity similarly in the two refractive groups, with both exhibiting significantly reduced and delayed b-wave amplitudes, which served as physiological proxies for retinal ON-responses. CMT changed after blur exposure in this experimental paradigm, although the change differed between the refractive groups: Myopic eyes showed a thinner muscle, whereas near-emmetropes developed a thicker CM. The additional exploratory ERG experiment revealed that defocus, when used in fixed-sequence order, reduced b-wave amplitudes in both groups under broadband and monochromatic red light conditions. Also, in both refractive groups, restriction to red light increased the b-wave implicit times, both without and with defocus.

Emmetropisation relates to the sensitive period of visually guided eye growth during the early postnatal years. As found in animal models, if positive or negative lenses are placed in front of the eye, then the retina locally detects the resulting blur and determines the sign of defocus to adjust the axial growth accordingly [1]. Processing of positive and negative defocus each activates different genes and pathways [47]. While some cues guiding refractive error development have been identified, the underlying principle of this active feedback loop is still being discussed [25, 48].

The use of ERG might increase current understanding of the retinal processing, which contributes to the conversion of the visual signal on the retina into molecular signals that ultimately lead to axial length changes. Such assessments could also provide insight into the changes in a myopic retina that might be responsible for an insufficient generation of inhibitory signals to guide eye growth. A previous study using multifocal ERG (mfERG) found that plus and minus lens defocus in young adults induced larger effects in the paracentral retina [26]. Applying pattern ERG, Panorgias et al. reported reduced retinal responses with simulated blur in emmetropes and myopes, especially in a 6–12° retinal area [49]. We recently confirmed in pattern ERGs that blur sensitivity depends on the retinal eccentricity, but also found an influence of the underlying refractive error [50]. In the present study, long flash ERGs were used to assess the effects of positive defocus on retinal ON vs. OFF pathways. Since this approach still does not allow for a clear separation of these two pathways, b- and d-wave amplitudes were used as physiological proxies for the retinal ON-/OFF-responses. While myopia has previously been suggested to be related to reduced ON pathway contributions [51], no significant differences were found between the proxies for retinal ON-/OFF-responses of myopes and near-emmetropes following longer plus lens wear in the present experimental paradigm. In both groups, a significant delay of ON-response proxies was observed after the blur experience of the fixed-order protocol. This is in contrast with an investigation using global-flash mfERGs in uncorrected eyes of adults with refractive errors between −6.00 and +4.00 D, in which retinal regions exposed to more hyperopic defocus showed larger amplitudes, while the response implicit times remained unaffected [52]. The rationale of Turnbull et al. for studying uncorrected eyes was to avoid possible lens-induced artefacts, such as aberrations or spectacle magnification [52]. However, the question arises whether retinal responses to the eye’s inherent refractive error can be compared with contact-lens corrected eyes following the imposition of positive lens defocus, as in the present work.

Previous axial length studies in young adults suggested a deficiency in myopic eyes to identify positive defocus and use chromatic information to guide ocular growth changes to defocus [17, 24]. This exploratory study of ERG components after exposure to defocus under broadband vs. red-light restriction, following a fixed-order protocol, did not reveal significant differences between the refractive groups. Red-light restriction in the sub-cohort induced reduced b-wave amplitudes after blur in both near-emmetropes and myopes, as was also found with broadband light. Further, in long wavelength light, b-waves were delayed in both refractive groups without and with blur. These increased latencies could derive from suppressed L-cone activity through adaptation mechanisms [53, 54] or from reduced inhibitory centre–surround interactions in spectrally opponent RGCs [55]. It should be noted that the present ERG endpoints might not have been sensitive to the tested conditions, otherwise retinal illuminance changes could have confounded the results. However, independent of the light spectrum, myopic participants of the exploratory study showed reduced retinal activity in the area of the i_off_-wave following the fixed-sequence exposure to plus lenses. This particular part of the ERG was previously found to be increased in patients with glaucoma [43] and optic neuropathy [55]. The present findings in the myopic sub-cohort could point to an imbalance of their retinal ON/OFF system [56, 57] or different inner retinal processing of defocus, requiring further assessment, preferably in a paediatric study group.

In addition, studying changes of the retinal ON/OFF system in myopes vs. emmetropes when placing negative lenses in front of the eye would be of interest: A recent trial in children compared the myopia control effects of a noncoaxial lens, providing a central zone for distance correction and a peripheral hexagonal lenslet array of either +3.00 or −3.00 D as a control area. Surprisingly, after 1 year, children wearing plus- or minus-powered lenslets showed significantly smaller myopia progression rates than the single-vision lens-wearing control group [58]. This approach contradicts findings in animal models, where negative lenses, by creating hyperopic defocus, induced enhanced eye growth and myopia [1]. Su et al. suggested that the lenslet array may have induced a phase delay of the wavefront, leading to increased higher-order aberrations at all viewing distances, so that the retina would be constantly exposed to a blurred image [58]. The treatment success with minus-powered lenslets might likewise be based on central–peripheral interactions or an adaptation to reduced peripheral contrast. These interactions could be studied in humans using visual electrophysiology. However, ERGs during negative lens wear would have to be recorded under cycloplegia to prevent compensatory accommodative changes, which limits the range of applicable ERG methods.

In the present study, it was demonstrated that a vital component of the accommodation system, the CM, was sensitive to the blur experience of the fixed-order protocol. The changes in CMT were used as a physiological proxy to assess potential adaptation to positive defocus. Interestingly, the CM reacted differently in the two refractive groups after 30 min of fixed-sequence plus lens wear. Myopic eyes showed significant thinning (around 20 µm) of the CM after 30 min of positive defocus, unlike near-emmetropic eyes. Hence, the CM in myopes underwent changes similar to those measured after a 30-min period of nearwork [36]. While still debated, nearwork might increase the risk of myopia development and progression [59]. It is plausible that sustained contraction of the CM during long periods of nearwork could induce retinal blur due to inadequate accommodation. This would be comparable to the blur resulting from positive lenses as used in the present investigation. Many optical methods of myopia management use plus lens defocus in the periphery to inhibit axial elongation [60]. Both prolonged myopic defocus [61] and retinal ON-stimulation [32] trigger an increase in choroidal thickness, which may protect against further axial elongation. The present finding that 30 min of plus lens wear in a fixed-sequence focus-defocus order induced the same change in CM morphology as a 30-min period of nearwork requires further assessment with respect to using plus lens defocus for myopia control. Moreover, it was shown that wearing plus lenses for only 30 min, using a fixed-order paradigm, led to significant understimulation of retinal ON pathways in both near-emmetropes and myopes, as seen from reduced b-wave amplitudes. These results raise the possibility that defocus-induced retinal activity changes contribute to the variability observed in optical treatment responses. Adequately designed paediatric longitudinal studies are needed to explore this hypothesis further.

A major internal-validity limitation of the present study is the fixed (not counterbalanced) sequence of experimental conditions (without defocus followed by with defocus), which might have affected the outcomes. Contributions from time-dependent adaptation, repeated testing or order effects to the described physiological changes cannot be excluded. Furthermore, the secondary outcome (use of red filters) is exploratory and remains underpowered for group-specific conclusions due to the small sample size. It would be interesting to study whether the effects of the red filter are sustained when adding a control experiment with neutral density filters.

It should be acknowledged that the pointwise *t*-test analysis used to assess the ERG response changes over time did not include correction for multiple testing. TFCE identified significant timepoints at 37 ms in the ON-response proxy of both groups (and at 38 ms when using the red filter) as well as at 527 ms in the OFF-response proxy of myopes in the exploratory experiment. The fact that no extended temporal cluster was detected reflects the conservative nature of TFCE, whereby isolated timepoints accumulate insufficient cluster mass to constitute a robust cluster-level effect. However, temporally restricted differences should not necessarily be considered less relevant than effects extending over broader temporal intervals.

The use of soft contact lenses to correct the myopic participants during the trial may have introduced variability, such as due to changes in the tear film or lens centration. Additionally, small shifts in exposure to defocus because of individual variations in contact lens fit or vertex distance might have occurred. The true magnitude of retinal defocus in both refractive groups remains unknown, which could have influenced the measurements, although any residual refractive errors were likely to have been small. Likewise, axial length differences between the two groups could have affected the retinal image size. With the myopic participants falling in the mid to lower range of myopia, this likely had a very limited impact on the ERG outcomes. Further, the accommodative response of the subjects while watching the movie at a 2.00 m distance was not recorded. This viewing distance, chosen on the basis of the study by Swiatczak and Schaeffel [17], would be expected to produce an accommodative response of approximately 0.50 D. Since this stimulus was used during all sessions (without/with plus lenses), all measurements would have been affected equally, and thus, it does not confound the findings. Additionally, the pupil was not dilated during the experiment, and therefore, both the red filter and the positive defocus might have influenced the ERG data by changing the retinal illuminance with varying pupil size. However, for all conditions, pupil sizes were recorded immediately after watching the movie, i.e., before the ERG measurement. Differences in pupil size were minimal and therefore are unlikely to explain the effects of positive defocus and/or red filter on the retinal activity described above (experiment 1: without defocus 5.68 ± 1.02 mm; with defocus 5.56 ± 0.93 mm; experiment 2: without defocus 4.85 ± 1.12 mm; with defocus 4.82 ± 0.99 mm).

This study revealed that in both near-emmetropes and myopes, 30 min of adaptation to plus lenses using a fixed-order focus-defocus protocol reduced b-wave amplitudes, serving as proxies for retinal ON-responses, also under conditions of red-light restriction. However, the CMT changed in the opposite direction after experiencing plus lens defocus between myopes and near-emmetropes. Importantly, these findings show short-term changes following positive defocus in young adults. Further clinical trials in children are required to determine whether these physiological changes are related to ocular growth signals, the progression of paediatric myopia or the response to optical myopia control treatments.

## Data Availability

The data are available from the corresponding author upon reasonable request.
